# Reliability and validity of four cognitive interpretation bias measures in the context of social anxiety

**DOI:** 10.3758/s13428-024-02576-0

**Published:** 2025-01-07

**Authors:** Sascha B. Duken, Jun Moriya, Colette Hirsch, Marcella L. Woud, Bram van Bockstaele, Elske Salemink

**Affiliations:** 1 Department of Clinical Psychology, Utrecht University, Utrecht, the Netherlands; 2 Faculty of Sociology, Kansai University, Suita, Japan; 3 Department of Psychology, King’s College London, London, UK; 4 Center for Anxiety Disorders & Trauma, South London and Maudsley NHS Foundation Trust, London, UK; 5 Department of Clinical Psychology and Experimental Psychopathology, University of Göttingen, Göttingen, Germany; 6 Mental Health Research and Treatment Center, Faculty of Psychology, Ruhr-University Bochum, Bochum, Germany; 7 Department of Developmental, Personality and Social Psychology, Ghent University, Ghent, Belgium; 8 Research Institute of Child Development and Education, University of Amsterdam, Amsterdam, the Netherlands; 9 Centre for the Advancement of Research on Emotion, School of Psychological Science, University of Western Australia, Crawley, Australia

**Keywords:** Social anxiety, Interpretive bias, Cognitive bias, Psychometrics, Measurement

## Abstract

People with social anxiety disorder tend to interpret ambiguous social information in a negative rather than positive manner. Such interpretation biases may cause and maintain anxiety symptoms. However, there is considerable variability in the observed effects across studies, with some not finding a relationship between interpretation biases and social anxiety. Poor psychometric properties of interpretation bias measures may explain such inconsistent findings. We evaluated the internal consistency, test–retest reliability, convergent validity, and concurrent validity of four interpretation bias measures, ranging from more implicit and automatic to more explicit and reflective: the probe scenario task, the recognition task, the scrambled sentences task, and the interpretation and judgmental bias questionnaire. Young adults (*N* = 94) completed interpretation bias measures in two sessions separated by one week. Psychometric properties were poor for the probe scenario and not acceptable for the recognition task. The reliability of the scrambled sentences task and the interpretation and judgmental bias questionnaire was good, and they correlated highly with social anxiety and each other, supporting their concurrent and convergent validity. However, there are methodological challenges that should be considered when measuring interpretation biases, even if psychometric indices suggest high measurement validity. We also discuss likely reasons for poor psychometric properties of some tasks and suggest potential solutions to improve the assessment of implicit and automatic biases in social anxiety in future research.

## Introduction

Social anxiety disorder is a prevalent burden around the globe and can cause severe life impairments (Stein et al., 2017). Theoretical perspectives and gold-standard treatments for social anxiety disorder consider maladaptive cognitions as a core problem causing and maintaining social anxiety (Hirsch et al., 2006; MacLeod & Mathews, 2012; Plana et al., 2014). Specifically, people with social anxiety disorder process social information in a biased manner, often interpreting ambiguous or even benign social cues as harmful or threatening (Hirsch et al., 2016; Mathews & MacLeod, 2005; Plana et al., 2014; Woud, 2023). However, while a large body of research supports the important role of interpretation biases in social anxiety (Chen et al., 2020; MacLeod & Mathews, 2012), there are also studies that did not find a relationship between interpretation biases and social anxiety (Chen et al., 2020). Psychometric properties of the tasks and questionnaires that measure interpretation biases could explain such inconsistent results (De Schryver et al., 2016; LeBel & Paunonen, 2011; Parsons et al., 2019). Therefore, we investigated the reliability and validity of widely used interpretation bias measures: the probe scenario task (Mathews & Mackintosh, 2000; Salemink et al., 2007b), the recognition task (Mathews & Mackintosh, 2000; Salemink et al., 2007b), the scrambled sentences task (SST; Burnett Heyes et al., 2017; Wenzlaff & Bates, 1998), and the interpretation and judgmental bias questionnaire (IJQ; Voncken et al., 2003).

As a precondition to investigating the role of interpretation biases in social anxiety, it is necessary to quantify them with measures that have high *internal consistency*. That is, measures must consistently capture the bias of interest with as little measurement error as possible, because measurement precision represents an upper bound for correlations with other variables (Parsons et al., 2019; Vul et al., 2009).

While interpretation biases can be conceptualized as relatively stable, trait-like individual differences (Mathews & MacLeod, 2005), they may also change over time or due to psychological interventions (MacLeod & Mathews, 2012). To investigate changes in interpretation biases, and whether they can explain a reduction of anxiety symptoms, it is necessary to measure biases at multiple time points. Therefore, measures must have high *test–retest reliability*, so that differences between multiple assessments represent a true change of the bias and not measurement error over time.

Given that all measures aim at quantifying interpretation biases in social anxiety, we expect them to correlate with each other (*convergent validity*) and to correlate with social anxiety severity (*concurrent validity*). However, different interpretation bias measures might also assess partially different rather than the same processes because interpretation biases occur at different levels of processing, ranging from relatively fast and automatic to more conscious and subjective evaluations of social information. Relatedly, interpretation bias measures differ in their degree of explicitness (Chen et al., 2020; Würtz & Sanchez-Lopez, 2023). Due to these differences, it is possible that there is variation in the strength of associations between the measures. For instance, a meta-analysis indicated that there is a stronger relationship between anxiety symptoms and interpretation biases when the latter are investigated with subjective rather than objective measures (Chen et al., 2020). Given that interpretation biases may be multifaceted and occur at different processing stages, biases assessed with different measures may add up or interact to cause anxiety symptoms (Hirsch et al., 2006; Huppert et al., 2007). Therefore, we tested how much variance in anxiety symptoms the combination of all four bias measures could account for, and which measures accounted for unique variance in anxiety symptoms over and above the other measures (*unique associations*).

In the probe scenario task (Mathews & Mackintosh, 2000; Salemink et al., 2007b), participants read and imagine themselves in short ambiguous social scenarios. These scenarios end with a word fragment that resolves the ambiguity either positively or negatively. The difference in time it takes individuals to solve negative versus positive fragments quantifies their interpretation bias. The probe scenario task assesses implicit and automatic processes, as it does not ask participants to explicitly interpret social situations (Würtz & Sanchez-Lopez, 2023). While it is used regularly in research (e.g., Salemink et al., 2007a, b, 2014), its psychometric properties have not been evaluated.

The recognition task (Mathews & Mackintosh, 2000; Salemink et al., 2007b) includes automatic and reflective components. It consists of two phases. In phase 1, participants read ambiguous social scenarios that end with a word fragment. They are asked to complete the word fragments, but this does not resolve the ambiguity. In phase 2, participants are presented with four statements regarding the scenarios from phase 1. One represents a negative interpretation of the original scenario (i.e., negative target), and a second one represents a positive interpretation (positive target). Another positive (positive foil) and a negative (negative foil) statement are unrelated to the original scenario. Participants rate the similarity in meaning of each statement in relation to the meaning of the original scenario. Interpretational tendencies can be investigated per condition (i.e., separately analyzing similarity ratings for positive and negative targets) or as a difference score. Previous studies reported acceptable to good reliability for separate similarity ratings (Houtkamp et al., 2017; Ji et al., 2024). Psychometric properties of a difference score have not been reported. Interpretations assessed with the recognition task correlate with anxiety symptoms, indicating concurrent validity (Houtkamp et al., 2017; Salemink & Wiers, 2011).

In the SST (Burnett Heyes et al., 2017; Wenzlaff & Bates, 1998), participants are presented with six words in random order. They rearrange five words to form a grammatically correct sentence. Depending on which words are used, the resulting sentence represents a negative or a positive statement. The ratio of negatively to positively solved sentences quantifies a negative interpretation bias. Often, the SST involves a cognitive load to yield more automatic responses. The interpretive processes investigated with the SST can be considered in the middle of a continuum from implicit and automatic to explicit and reflective measures (Würtz & Sanchez-Lopez, 2023). The SST generally shows good reliability and correlations with psychopathological symptoms (e.g., a meta-analysis yielded reliability and correlation estimates of α = .79 and *r* = .46, respectively; Würtz et al., 2022). However, there is high heterogeneity in these estimates, and only a few studies investigated the SST in the context of social anxiety (e.g., Burnett Heyes et al., 2017). One study also reported a positive correlation between the interpretation bias scores based on the SST and the recognition task (de Voogd et al., 2017).

The IJQ (Voncken et al., 2003) consists of brief written social and nonsocial scenarios, based on evidence that interpretation biases in social anxiety are not generic but specific for the processing of social information (Butler & Mathews, 1987; Foa et al., 1996; McManus et al., 2000; Stopa & Clark, 2000). The social items include positive, ambiguous, mildly negative, and profoundly negative scenarios. Each scenario is followed by four possible interpretations ranging from positive to negative. Participants rank the interpretations in the order of their likelihood. The average rank of the likelihood of the most negative interpretation represents the tendency to evaluate social situations negatively. The IJQ can be considered an explicit measure of reflective processes with high internal consistency (Voncken et al., 2003). Individuals with social phobia rate negative interpretations to be more likely than individuals without social anxiety, indicating concurrent validity (Voncken et al., 2003, 2007).

In sum, there are differences in how thoroughly the psychometric properties of different approaches to measuring interpretation biases are understood. We conducted a preregistered lab-based study in which participants completed four social anxiety-related interpretation bias measures twice, with one week in between. We expected at least adequate internal consistency and test–retest reliability for each measure. We also expected correlations between the measures and with anxiety symptoms indicating convergent and concurrent validity, respectively. Moreover, we assessed whether each measure explained unique variance in social anxiety.

## Methods

### Participants and general procedure

A total of *N* = 100 participants started Session 1. Six participants were excluded (*n* = 3 for not attending Session 2, *n* = 3 due to experimenter mistakes), resulting in a sample of *N* = 94 participants. For some participants, data for specific tasks had to be excluded (see Data Analysis and Appendix). Originally, we aimed for a sample size of *N* = 100 participants after exclusion, likely to result in confidence intervals for the intraclass correlation coefficients (ICCs) with a width of .30 or less, and a power of .80 to detect correlations of .30 (see preregistration: 10.17605/OSF.IO/SF6ZV).

Participants were recruited through the study participation web portal of Utrecht University as well as through flyers and posters on campus. Participants were on average 22.40 years old when completing Session 1 (*SD* = 4.21, range = 17–44). Seventy-seven participants reported being female, 17 reported being male. Session 2 was scheduled 6 to 8 days after Session 1, but sometimes it had to be rescheduled, for example, due to illness. The average time between sessions was 7.27 days (*SD* = 1.38, range = 6–14). All participants spoke Dutch on a “mother tongue” level, except two who spoke Dutch “very well.”

Two lab sessions were conducted in the behavioral sciences laboratory of Utrecht University. Upon arrival for Session 1, participants were informed about the general procedure and provided written informed consent. They were seated alone in front of a computer screen in a small room. The experimenter was available in an adjacent room. Participants received all instructions on screen, unless stated otherwise. All instructions and materials were in Dutch. Participants first responded to demographic questions and then completed the probe scenario task, the two phases of the recognition task, the SST, and the IJQ. Finally, participants completed the Social Interaction Anxiety Scale (SIAS; de Beurs et al., 2014). All participants completed all tasks in the same order to reduce error variance across participants due to the order of tasks (De Schryver et al., 2016; Perugini et al., 2010). The procedure for Session 2 was identical to the first, except that participants did not provide new written consent. The specific stimuli in Session 2 for the probe scenario task, recognition task, and SST were different from Session 1. Some variability across measurements may therefore result from the difference in the task versions (e.g., when assessing test–retest reliability). However, we assumed that the variance due to different task versions should be negligible, given that different studies often employ different stimuli in random order and assume that the different task versions measure similar interpretive processes. Notably, it is not possible to use the same stimuli twice for a participant, because once an item is known and the respective interpretation has occurred, a person does not need to interpret the stimulus anew but can rely on the previously made interpretation. The items of the IJQ and SIAS questionnaires were the same in both sessions. Participants received compensation with partial course credits or a small amount of money. The study protocol was approved by the ethical committee of Utrecht University (FETC 18–132). Analyses were preregistered on the Open Science Framework (OSF) while data collection was ongoing but before data was analyzed by any of the responsible researchers (10.17605/OSF.IO/SF6ZV). Materials to which no legal restrictions apply, data, and analysis scripts are available on OSF [database] (Duken et al., 2024). A subsample was analyzed in student projects prior to preregistration.

### Measures and tasks

#### Probe scenario task

Participants read and imagined themselves in 20 short ambiguous social scenarios that ended with a word fragment (Salemink et al., 2007b). Half of the word fragments resolved the ambiguity positively, and half of them resolved it negatively, if completed correctly. Each scenario was presented with three lines of text in white font that appeared on a black screen. Each line of text was presented for 2.5 s before the next line appeared below it. The word fragment appeared below 2.5 s after all lines were visible (e.g., first line: “*You spent the evening with a friend and*”; second line: “*you end up talking about his relationship problems. You expect that ….*” third line: “*he will find your advice ….*”; word fragment: “*use_ _ll*”). Participants were asked to press the spacebar as soon as they knew the complete word, and to type the word using the keyboard. Reaction times were measured from the moment the word fragment appeared to the moment participants pressed the spacebar. Then, they responded to a comprehension question by clicking “yes” or “no” with the mouse or by pressing the numbers 1 or 2 on the keyboard (e.g., “*Did your friend think that you gave good advice about his relationship?*”). Participants received feedback on the comprehension question with the words “*Correct!*” or “*Incorrect!*” on screen. Instructions and two practice trials were provided before participants started the task. The order of the statements was randomized. We calculated an interpretation bias index by subtracting the mean reaction time to correctly solved positive scenario trials from the mean reaction time to correctly solved negative scenario trials per participant. The lower the difference score, the faster a participant responded to negative scenario trials compared to positive scenario trials, indicating that they more readily made negative interpretations. In secondary analyses, we also analyzed reaction times in positive and negative trials separately.

#### Recognition task

The recognition task consisted of two phases (Salemink et al., 2007b). In phase 1, participants read ambiguous social scenarios that also ended with a word fragment, similar to the probe scenario task. However, the scenarios remained ambiguous after completing the word fragment. Additionally, each scenario in phase 1 of the recognition task started with a title. The title was displayed for 2 s, before the three lines of text and the word fragment appeared sequentially, with 2.5 s in between (e.g., title: “*Your sister’s colleagues*”; first line: “*Your sister introduces you to her colleagues whom you have never met before*”; second line: “*You try to have a conversation with them*”; third line: “*and after a while you get an impression of how they … the conversation”*; word fragment: “*f_nd*”). Participants were again asked to press the space bar and to type the word as soon as they knew it, followed by a comprehension question with feedback. Instructions and two practice trials were presented on screen before the task started. The scenarios were selected from previous studies (Chow et al., 2018; Lothmann et al., 2011; Salemink et al., 2007b).

During phase 2 (of which participants had not been informed before), participants were presented with four possible interpretations for each of the scenarios from phase 1. There were two possible interpretations: one matched a negative interpretation of the original scenario (negative target; e.g., “*Your sister’s colleagues find the conversation annoying.*”), and one matched a positive interpretation (positive target; e.g., “*Your sister’s colleagues find the conversation fascinating.”*). There were also two unrelated foil statements: one was a negative statement (negative foil; e.g., “*You hope that your future colleagues will be less antisocial than your sister’s.*”), and one was a positive statement (positive foil; e.g., “*You hope that your future colleagues will be as nice as your sister’s.*”). Participants rated the similarity in meaning of each statement in relation to the meaning of the original scenario on a four-point scale, with 1 = *very different meaning*, 2 = *quite different meaning*, 3 = *quite similar meaning*, and 4 = *very similar meaning*. Each statement was presented individually together with the scenario title from phase 1. The order of the scenarios and the order of statements per scenario were randomized. The similarity rating was self-paced. We calculated an interpretation bias index by subtracting the similarity rating of the positive targets from the similarity rating of the negative targets per scenario. In secondary analyses, we also investigated psychometric properties of the ratings of positive and negative interpretations separately.

#### Scrambled sentences task (SST)

Participants received two sheets of paper with 10 scrambled sentences on each. Each scrambled sentence consisted of six words in an incoherent order (e.g., “*will people my mistakes notice talents*”). Participants rearranged five words to form a grammatically correct sentence, leaving the sixth word out. Depending on which words were used, the resulting sentence represented a negative (e.g., “*people will notice my mistakes*”) or positive statement (e.g., “*people will notice my talents*”). The scrambled sentences were Dutch translations of the originally English version of Burnett Heyes and colleagues (2017). To introduce a cognitive load, participants were asked to remember a six-digit number that they had to reproduce after solving 10 sentences. Participants read instructions and an example scrambled sentence on screen, but there was no practice trial. After this, they saw an instruction on screen to call the experimenter. The experimenter put the two sheets with the scrambled sentences face down on the table and started the task. Participants first saw a warning for 4990 ms that they would soon see the to-be-remembered code on screen. After another 10 ms, the six-digit number 914073 was presented for 5 s (the code in Session 2 was 078432). Ten seconds after the number disappeared, the instruction appeared that participants should solve the first page of scrambled sentences and press the letter “t” on the keyboard when they finished. Following, they were asked to type the digit number using the keyboard. This procedure was repeated for the second page of the SST with the numbers 257190 and 495801 in Session 1 and 2, respectively. The proportion of negatively solved sentences among all grammatically correct negative and positive solutions quantified participants’ negativity bias.

#### Interpretation and judgmental bias questionnaire (IJQ)

The interpretation and judgmental bias questionnaire (Voncken et al., 2003) consists of brief written social and nonsocial scenarios. The social items include positive, ambiguous, mildly negative, and profoundly negative scenarios. Each scenario is followed by four possible interpretations ranging from positive to negative. Participants rank the interpretations in the order of their likeliness from most likely (1) to least likely (4). This ranking was reverse-scored so that higher numbers reflect higher likelihood. The IJQ was administered as a pen-and-paper questionnaire. The mean rank of the likelihood of the most negative interpretation quantified an individual’s tendency to evaluate social situations negatively. We also calculated the mean rank of the profoundly positive interpretation for secondary analyses.

#### Social Interaction Anxiety Scale (SIAS)

Social anxiety symptoms were measured with the Dutch version of the SIAS (de Beurs et al., 2014) consisting of 20 statements, such as “*I get nervous if I have to speak with someone in authority (teacher, boss, etc.).*” Participants indicated how characteristic each statement was for them in the past week on a scale with 0 = *not at all*, 1 = *slightly*, 2 = *moderately*, 3 = *very*, and 4 = *extremely*. The SIAS has high internal consistency as well as good concurrent and discriminant validity (de Beurs et al., 2014). SIAS scores range between 0 and 80, with higher scores reflecting more severe social anxiety symptoms. Psychometric properties and descriptive statistics for the SIAS in this study are presented in Table 1.

**Table 1:** Descriptive statistics, internal consistency, and test–retest reliability estimates

		Session 1			Session 2			Test–retest reliability		
		*M* (*SD*)	Split-half [CI]	α	*M* (*SD*)	Split-half [CI]	α	ICCa [CI]	ICCc [CI]	*r*/*ϱ* (*p*)
Probe scenario task	Difference	241.7 (479.6)	−.42 [−.72, .03]	-	97.1 (521.8)	.28 [−.43, .68]	-	.00 [−.19, .20]	.00 [−.20, .20]	*r* = −.236 (.986)
	Negative	2205.0 (1889.4)	.95 [.90, .97]	.96	1512.6 (1627.9)	.97 [.94, .98]	.97	.79 [.48, .89]	.85 [.78, .89]	*r* = .731 (< .001)
	Positive	1963.3 (1628.0)	.92 [.87, .96]	.94	1415.5 (1564.4)	.97 [.94, .98]	.98	.81 [.60, .90]	.85 [.79, .90]	*r* = .681 (< .001)
Recognition task	Difference	−0.22 (0.638)	.44 [.25, .59]	.43	−0.25 (0.540)	.30 [.08, .49]	.32	.26 [.07, .44]	.26 [.07, .44]	*r* = .002 (.492)
	Negative	2.33 (0.485)	.48 [.32, .62]	.48	2.36 (0.456)	.60 [.48, .70]	.60	.45 [.27, .60]	.45 [.27, .60]	*r* = .445 (< .001)
	Positive	2.55 (0.365)	.07 [−.17, .31]	.06	2.61 (0.359)	.21 [−.04, .42]	.21	.32 [.13, .49]	.32 [.13, .49]	*r* = .322 (.001)
SST	Ratio	.2202 (.1762)	.75 [.67, .83]	.80	.2099 (.1898)	.80 [.73, .86]	.82	.85 [.79, .90]	.85 [.79, .90]	*ϱ* = .826 (< .001)
IJQ	Negative	1.46 (0.41)	.82 [.75, .87]	.86	1.37 (.38)	.84 [.78, .89]	.87	.86 [.73, .92]	.88 [.83, .92]	*ϱ* = .834 (< .001)
	Positive	2.51 (0.43)	.79 [.73, .85]	.83	2.56 (0.44)	.82 [.77, .87]	.85	.88 [.82, .92]	.89 [.83, .92]	*ϱ* = .887 (< .001)
SIAS	Sum	23.06 (13.81)	.91 [.88, .94]	.93	22.88 (14.18)	.92 [.90, .94]	.94	.94 [.90, .96]	.94 [.90, .96]	*r* = .935 (< .001)

Data are presented for the probe scenario task, recognition task, scrambled sentences task (SST), interpretation and judgmental questionnaire (IJQ), and Social Interaction Anxiety Scale (SIAS). *M* = mean; *SD* = standard deviation; CI = confidence interval; α = Cronbach’s alpha; ICCa = intraclass correlation (agreement), ICCc = intraclass correlation (consistency); *r* = Pearson’s correlation; ϱ = Spearman’s correlation; *p* = *p*-value

#### Demographic questions

In both sessions, participants self-reported their sex as “*woman*” or “*man*,” how old they were in years, and how fluently they spoke Dutch (with 1 = *mother tongue*, 2 = *very well*, 3 = *reasonably well*, 4 = *mediocre*, and 5 = *not so well*).

### Data analysis

All analyses were conducted in RStudio (Posit Software, 2023; R Core Team, 2023). Reliability indices were considered acceptable if they were .7 or higher, good if .8 or higher, and very good if .9 or higher. For validity analyses, the significance level was set to α = .0125 to correct for multiple comparisons.

#### Internal consistency

We estimated the internal consistency of the four interpretation bias measures using permutation-based split-half correlations with 5000 random splits, corrected with the Spearman–Brown prophecy formula using the *splithalf* package (Parsons, 2021; Parsons et al., 2019). The internal consistency was evaluated separately for both sessions, with results from Session 1 being decisive and the Session 2 providing additional information. For completeness and consistency with previous work (e.g., Voncken et al., 2003), we also report Cronbach’s alpha.

#### Test–retest reliability

We quantified test–retest reliability using the intraclass correlation coefficient (ICC) for consistency and agreement using the *psych* package (Revelle, 2023). Consistency quantifies the extent to which scores are in the same order or pattern at multiple assessments. Agreement quantifies how similar scores are across assessments. For instance, if a group of participants scores systematically higher in a second than in a first assessment, the scores are different (low agreement), but the pattern across participants may be preserved (high consistency). If consistency is high but agreement is low, a measure is well suited for investigating individual differences but not change in individuals over time. If consistency and agreement are high, a measure is well suited for investigating individual differences and change in individuals. We also report correlations between the measures in both sessions.

#### Convergent and concurrent validity

To evaluate convergent validity, we calculated Spearman correlations (visual inspection of the data suggested that some were right-skewed, in particular the SST and IJQ). To evaluate concurrent validity, we calculated the Spearman correlation of each measure with social anxiety symptoms assessed with the SIAS.

#### Unique associations with anxiety

We examined whether the four interpretation bias measures were uniquely associated with social anxiety levels in a multiple regression analysis. If a measure explained variance beyond the other measures, this would indicate that it captured a distinct aspect or level of interpretation bias.

#### Data exclusion

Prior to calculating any indices, we excluded invalid data points on the item level. In the probe scenario task and the recognition task, trials in which a participant entered an incorrect word were considered invalid (words with a typo that were clearly understandable and that did not form a different existing Dutch word were not considered invalid). Trials with a response time below 200 ms were also excluded. Finally, we removed outliers for the reaction times using the median absolute deviation procedure per individual, task, and session with the threshold 2.5 (Leys et al., 2013). In the SST, trials were excluded when participants did not create a correct sentence that was either positive or negative.

We excluded data on the participant level for an entire task per session if they did not meet basic quality checks. For the probe scenario and the recognition task, we excluded data from participants who performed three standard deviations or more below the sample mean on the comprehension question, if they also answered less than 75% of the questions correctly. These cutoffs were chosen a priori to exclude participants who did not engage with the task as a whole, while including participants who did engage with the task but did not perform well. For the SST, only participants who provided a grammatically correct solution for at least half of the trials in the SST were included. For the IJQ, participants were excluded if they did not provide correct responses to at least half of the items. A summary of excluded trails and participants per task can be found in the Appendix.

## Results

Descriptive statistics, internal consistency estimates, and test–retest reliability estimates for all measures are presented in Table 1. SIAS scores in Session 1 ranged between 2 and 59 (25th percentile = 11, 50th percentile = 19.5, 75th percentile = 32).

### Internal consistency and test–retest reliability

#### Probe scenario task

In Session 1, participants needed on average 2.2 s to resolve negative fragments and 1.9 s to resolve positive fragments (Table 1). In Session 2, they needed 1.5 and 1.4 s for negative and positive fragments, respectively. The Spearman-Brown-corrected estimate for the internal consistency of the difference between the reaction time to negative and positive fragments was negative for Session 1 and very low for Session 2. The difference score also showed no test–retest reliability. The reliability of reaction times assessed separately for negative and positive trials was high in Session 1 and in Session 2. Reaction times to negative and positive trials assessed separately showed high test–retest reliability.

#### Recognition task

In Session 1, the average similarity rating was 2.3 for negative targets and 2.6 for positive targets (Table 1). In Session 2, the similarity ratings were 2.4 and 2.6 for negative and positive targets respectively. The internal consistency of the difference between the similarity rating of negative and positive targets was not acceptable in Session 1 and Session 2. The test–retest reliability was low.

The internal consistency of similarity ratings of positive targets alone was also not acceptable. The test–retest reliability of positive targets was low, even though there was a significant positive correlation between Session 1 and Session 2 ratings of positive targets. The internal consistency of similarity ratings of negative targets was better than for positive targets but still not acceptable. The test–retest reliability of similarity ratings of negative targets was also low, but there was a significant positive correlation between Session 1 and Session 2 ratings.

#### Scrambled sentences task

In Session 1 and 2, 22% and 21% of correct sentences were negatively solved. The internal consistency of the SST was good in Session 1 and in Session 2. The test–retest reliability was high. Data regarding the retention of the digit number and trial exclusions due to incorrect responses or short reaction times are presented in the Appendix.

#### Interpretation and judgmental questionnaire

The negative interpretations were on average ranked between least likely and second least likely in both sessions (rank 1.5 among four possible ranks). The average rank of positive interpretations was 2.5 and 2.6 in Session 1 and 2, respectively. The internal consistency of the rank of the negative interpretation was good in Session 1 and Session 2. The test–retest reliability was high. The internal consistency of the rank of positive interpretations was also good, with high test–retest reliability.

### Convergent and concurrent validity

Table 2 shows Spearman correlations between each interpretation bias and with social anxiety. In Session 1, the SST, IJQ, and SIAS correlated significantly and highly with each other, but not with the probe scenario or the recognition task. In Session 2, the SST, IJQ, and SIAS were again highly correlated. In contrast to Session 1, the recognition task in Session 2 correlated significantly with the SST, IJQ, and SIAS.

**Table 2:** Correlations between interpretation bias measures and social anxiety

	Probe scenario	Recognition	SST	IJQ	SIAS
Probe scenario task	–	.057 (.296)	.038 (.363)	.052 (.314)	.206 (.026)
Recognition task	.147 (.084)	–	.127 (.118)	.060 (.288)	.173 (.051)
SST	.126 (.120)	.425 (< .001)*	–	.694 (< .001)*	.673 (< .001)*
IJQ	.051 (.317)	.408 (< .001)*	.679 (< .001)*	–	.670 (< .001)*
SIAS	.007 (.472)	.322 (.001)*	.715 (< .001)*	.598 (<.001)*	–

The upper right half of the table presents correlation coefficients from Session 1, the lower left half from Session 2. Each cell contains the Spearman correlation coefficient and the corresponding one-sided *p*-value in brackets below. Results are presented for the primary interpretation bias indices (the first row per measure in Table 1). To account for multiple comparisons, the significance level for these analyses was set to α = .0125. Significant correlations are indicated with an asterisk (*)

#### Unique association of the interpretation bias measures with social anxiety

We investigated whether the four interpretation bias measures were uniquely associated with social anxiety symptoms, and how much unique variance each interpretation bias measure accounted for, separately for both sessions. We conducted multiple regression analyses with SIAS score as the dependent variable and the four interpretation bias measures as independent variables. All variance inflation factors were below 2.5, indicating that model estimates were not affected by multicollinearity (Petrie, 2020).

In Session 1, the interpretation bias measures accounted for 55% of the variance in social anxiety symptoms (*F*(4,84) = 25.76, *p* < .001). Only the SST (*b* = 32.735, *SE* = 8.499, *t* = 3.852, CI [15.834, 49.635], *p* < .001, β = 0.420) and the IJQ (*b* = 10.661, *SE* = 3.651, *t* = 2.920, CI [3.401, 17.921], *p* = .004, β = 0.317) were uniquely associated with anxiety symptoms. The slopes of the probe scenario task (*b* = 4.468, *SE* = 2.250, *t* = 1.986, CI [−0.005, 8.942], *p* = .050, β = 0.148) and the recognition task (*b* = 1.191, *SE* = 1.610, *t* = 0.740, CI [−2.012, 4.395], *p* = .462, β = 0.055) were not significant. Investigated separately in regressions with only one independent variable, the SST accounted for 48% of the variance of social anxiety symptoms, and the IJQ accounted for 44%.

In Session 2, the interpretation bias measures accounted for 49% of the variance of social anxiety symptoms (*F*(4,82) = 21.69, *p* < .001). Only the SST was uniquely associated with social anxiety symptoms (*b* = 43.575, *SE* = 9.041, *t* = 4.820, CI [25.589, 61.560], *p* < .001, β = 0.586). The slopes of the IJQ (*b* = 4.121, *SE* = 4.585, *t* = 0.899, CI [−2.165, 6.681], *p* = .371, β = 0.107), the probe scenario task (*b* = 0.780, *SE* = 1.914, *t* = 0.418, CI [−3.007, 4.606], *p* = .677, β = 0.033), and the recognition task (*b* = 2.257, *SE* = 2.223, *t* = 1.015, CI [−2.165, 6.681], *p* = .313, β = 0.087) were not significant. Investigated separately in regressions with only one independent variable, the SST accounted for 51% of the variance in social anxiety symptoms, and the IJQ accounted for 28%. Unique associations between interpretation measures in Session 1 and social anxiety symptoms in Session 2 are presented in the Appendix.

## Discussion

We investigated the internal consistency, test–retest reliability, and validity of interpretation bias measures across two sessions with one week in between. Psychometric properties of the probe scenario task and the recognition task were poor. The SST and the IJQ had good psychometric properties and high correlations with each other as well as with social anxiety symptoms.

The difference score in the probe scenario task was not internally consistent, had low test–retest reliability, and did not correlate with social anxiety or other interpretation bias measures. Reaction times in negative and positive trials investigated separately were reliable, but such scores cannot be used to infer interpretation biases without further processing, as the variance is mostly determined by an individual’s general response speed. These results are in line with research demonstrating low reliability of other implicit measures of automatic cognitive biases that rely on reaction times (Brown et al., 2014; LeBel & Paunonen, 2011; O’Connor et al., 2021). One explanation may be that bias measures derived from reaction time tasks often rely on difference scores between positive and negative trials (LeBel & Paunonen, 2011). Cognitive processes that lead to positive or negative interpretations may be qualitatively different from each other rather than unidimensional (Huppert et al., 2003; Steinman et al., 2020). In that case, a difference score can conflate information from different processes and yield low or even negative reliability estimates (Cronbach & Hartmann, 1954). Future research could investigate avenues to capture automatic biases by investigating positive and negative interpretations separately. One solution may be to include neutral control trials to investigate the difference between positive versus neutral trials, and negative versus neutral trials. In any case, the probe scenario task should not be used further to investigate interpretation biases in anxiety without a substantial revision and psychometric evaluation.

The recognition task yielded numerically higher reliability estimates than the probe scenario task (even though most confidence intervals overlapped), but neither its internal consistency nor test–retest reliability were acceptable. The recognition task correlated moderately with anxiety as well as with the SST and the IJQ in Session 2, but not in Session 1. This might indicate that familiarity with the task improves its validity. However, the internal consistency was not better in Session 2. The poor psychometric properties of the recognition task are in conflict with studies that reported acceptable internal consistency and correlations with anxiety (Houtkamp et al., 2017; Ji et al., 2024). It is possible that the scenarios employed in this study require improvement, while other versions of the recognition task are reliable and valid.

In general, poor psychometric properties may result from a suboptimal selection of stimuli, rather than a task in general. Specifically, for many interpretation bias measures (including the probe scenario task, the recognition task, and the SST), stimuli often vary across studies, and there is limited information available on stimulus characteristics such as word or scenario length, difficulty, or likelihood of occurrence. It may be possible to develop stimulus sets that include such information and allow researchers to control for confounding variables (statistically or by matching stimuli across conditions and individuals), thereby increasing reliability. As long as such stimulus sets are not available, it is important to either pilot or at least carefully evaluate and report psychometric properties of the specific task versions that are used.

The SST and the IJQ had good psychometric properties and correlated highly with social anxiety and each other, in line with previous research (Burnett Heyes et al., 2017; Voncken et al., 2003; Würtz et al., 2022). These findings suggest that both measures represent strong tools to investigate interpretation biases in social anxiety. Moreover, different versions of the SST are able to capture interpretation biases that are relevant in other disorders than social anxiety, for example depression and posttraumatic stress disorder (O’Connor et al., 2021; Würtz et al., 2022). The SST may therefore be a valuable tool to investigate biases across mental disorders. The SST version that we evaluated is, however, not able to assess negative and positive interpretations separately. If negative and positive interpretations rely on different processes, it may be worth developing SST versions in which the solved sentences are negative versus neutral or positive versus neutral statements (instead of negative versus positive).

Next to the psychometric properties investigated in this study, there are other important methodological considerations when designing studies on interpretation biases and anxiety. First, items of interpretation bias measures may load not only on latent interpretation biases, but also on anxiety symptoms that the biases are supposed to explain. For instance, the SIAS item “*I find it difficult to talk with people*” and the SST item “*Talking with people is difficult/easy*” are very similar. If interpretation bias measures aim to explain variance in social anxiety, such overlap in measurement should be avoided. In correlational studies, factor analyses may help to ensure that items of bias measures do not also load on anxiety measures. Second, explicit and reflective measures bear a high risk for social desirability and other response biases (LeBel & Paunonen, 2011). To some extent, this may also apply to measures that are in between explicit and implicit. For instance, while completing the SST, participants are likely to notice that unscrambled sentences are either negative or positive self-referential statements, and may try to provide solutions in line with their self-image or with perceived study demands. For explicit measures, people also must be aware of the cognitive processes under investigation. However, not all cognitive processes that cause and maintain anxiety may be accessible to one’s awareness (Teachman et al., 2012), and an individual’s degree of self-awareness may be a cognitive vulnerability factor in itself. Moreover, some people may experience a bias in reflective processes while others may experience a bias in automatic processes. Therefore, explicit and implicit measures are needed to investigate the full range of interpretative processes that cause and maintain anxiety. While explicit measures show good psychometric properties, it is necessary to develop new or improve existing implicit measures. O’Connor and colleagues (2021) found a similar pattern when investigating interpretation bias measures in the context of depression. They reported poor psychometric properties of indirect and good properties of direct measures, and only the SST uniquely explained variance in depressive symptoms. This suggests that limitations of implicit interpretation bias measures extend beyond research on social anxiety.

The current study has several limitations. First, the sample size was relatively small for a psychometric study. However, the results were consistent across analyses (e.g., split-half and α) and sessions with only a few exceptions (the recognition task correlated with other measures in session 2 but not in session 1, the IJQ did not explain unique variance in anxiety symptoms over and above the SST in session 2). Based on the clear pattern of results, we assume that our conclusions are warranted by the data and results. Second, we could only include a selection of tasks that are relevant to understanding social anxiety symptoms. Generalizations to other measures or anxiety problems are not warranted. Moreover, the materials were in Dutch. Tasks using stimuli in other languages should also be investigated. Nonetheless, our results indicate that future research needs to more carefully evaluate and report psychometric properties of employed measures, especially when investigating automatic biases. Third, we evaluated psychometric properties based on a priori defined analysis decisions, but there is a multitude of analysis options that can influence the psychometric properties of interpretation bias measures (Parsons, 2023). However, we consider it unlikely that any reasonable deviation from our analysis plan would result in acceptable psychometric properties for the probe scenario or the recognition task. Finally, our sample consisted of unselected students who self-reported their social anxiety symptoms. It is possible that the employed measures show different psychometric properties in samples with diagnosed anxiety disorders (Maassen et al., 2023). However, social anxiety symptoms are common among students (Jefferies & Ungar, 2020; Russell & Shaw, 2009), and our sample included a wide range of symptoms, including potentially clinical levels. Assuming social anxiety symptoms to be situated on a continuum from absent to clinical, we consider our sample well suited to evaluate interpretation biases and social anxiety.

In sum, the SST and the IJQ had good psychometric properties and can represent strong tools to investigate interpretive processes in social anxiety, if other methodological challenges are considered carefully (e.g., stimulus choice and the degree to which a bias can be reported on accurately). The probe scenario task and the recognition task had poor psychometric properties and should not be used in their current form. Our study further underscores the need to investigate and report psychometric properties of the measures that we use to investigate the mechanisms that underlie anxiety symptoms.

## Data Availability

Data and materials to which no legal restrictions by third parties apply are publicly available on the Open Science Framework (Duken et al., 2024; 10.17605/OSF.IO/Y7346).

## Appendix

### Probe scenario task

Probe Scenario Task data was available for 90 participants in session 1. Data for 92 participants was available in session 2, but one was excluded because they did not give sufficient correct responses to the comprehension questions (resulting in N = 91). For session 1, 193 trials were excluded from a total of n = 1800 (n = 20 incorrect responses, n = 5 response times below 200ms, n = 168 outliers). For session 2, 240 of 1820 trials were excluded (n = 37 incorrect responses, n = 5 response times below 200 ms, n = 198 outliers).

### Recognition task

Recognition Task data was available from 90 participants in session 1. Data was available from 92 participants in session 2, but one was excluded because they responded incorrectly to three out of eight comprehension questions (resulting in *N* = 91). For session 1, 4 of 720 trials were removed (*n* = 2 incorrect responses, *n* = 2 response times below 200 ms). There were *n* = 81 outliers in terms of reaction times in phase 1, but those data were not excluded given that the phase 2 similarity ratings were the variable of interest. For session 2, 73 of 728 trials were removed (*n* = 2 incorrect responses, *n* = 4 reaction times below 200 ms, There were *n* = 67 outliers in terms of reaction times in phase 1, but those data were not excluded.

### Scrambled sentences task

SST data were available from 93 participants in the first session and 92 participants in the second session. 42 of 1860 trials and 38 of 1840 trials were excluded from the session 1 and session 2 data, respectively, because they were not correctly solved in time or not clearly positive or negative.

Data regarding the reproduction of the digit number that represented a cognitive load was available for 89 participants in session 1 and 90 in session 2 (only among participants that otherwise had valid SST data), respectively. In session 1, eleven out of 89 participants did not reproduce at least one number correctly. In session 2, four out of 90 participants did not reproduce at least one number correctly. Only one participant did not reproduce any of the four digit numbers across two sessions correctly, and also that participant almost reproduced the correct numbers (e.g., 934170 and 078324 instead of 914073 and 078432). Overall, we conclude that participants adhered to the instructions and attempted to keep the digit number in mind, even though they did not always succeed in reproducing it correctly.

### Associations between interpretation bias measures in session 1 and anxiety symptoms in session 2

We conducted an exploratory multiple regression to investigate the unique associations of interpretation bias measures in Session 1 and social anxiety symptoms in Session 2. The interpretation bias measures in session 1 accounted for 52% of the variance in social anxiety symptoms in session 2 (*F*(4,84) = 24.62, *p* < .001). Only the SST (*b* = 37.916, *SE* = 8.853, *t* = 4.283, CI[20.311, 55.521], *p* < .001, *β* = 0.473) and the IJQ (*b* = 9.756, *SE* = 3.803, *t* = 2.565, CI[2.193, 17.318], *p* = .012, *β* = 0.282) were uniquely associated with anxiety symptoms in the next session. The slopes of the Probe Scenario Task (*b* = 2.600, *SE* = 2.343, *t* = 1.110, CI -2.059, 7.260], *p* = .270, *β* = 0.084) and the Recognition Task (*b* = 0.830, *SE* = 1.678, *t* = 0.495, CI[-2.507, 4.167], *p* = .622, *β* = 0.037) were not significant. Investigated separately in regressions with only one independent variable, the SST accounted for 50% of the variance of social anxiety symptoms, and the IJQ accounted for 42%.

## Code availability

All analysis code is publicly available on the Open Science Framework (Duken et al., 2024; 10.17605/OSF.IO/Y7346).
